# Preparation of Nanoscale Urushiol/PAN Films to Evaluate Their Acid Resistance and Protection of Functional PVP Films

**DOI:** 10.3390/nano11040957

**Published:** 2021-04-09

**Authors:** Kunlin Wu, Bing-Chiuan Shiu, Ding Zhang, Zhenhao Shen, Minghua Liu, Qi Lin

**Affiliations:** 1 Fujian Engineering and Research Center of New Chinese Lacquer Materials, Ocean College, Minjiang University, Fuzhou 350108, China; Kunlinwu2020@163.com (K.W.); bcshiu@mju.edu.cn (B.-C.S.); dingzhang1874@126.com (D.Z.); s951623874@126.com (Z.S.); 2 College of Environment and Resources, Fuzhou University, Fuzhou 350108, China; mhliu2000@fzu.edu.cn; 3 College of Materials Science and Engineering, Fuzhou University, Fuzhou 350108, China

**Keywords:** urushiol, polyacrylonitrile (PAN), electrospinning, acid membrane, nanofibers

## Abstract

Different amounts of urushiol were added to a fixed amount of polyacrylonitrile (PAN) to make nanoscale urushiol/PAN films by the electrospinning method. Electrospinning solutions were prepared by using dimethylformamide (DMF) as the solvent. Nanoscale urushiol/PAN films and conductive Poly(3,4-ethylenedioxythiophene):poly(styrenesulfonate)(PEDOT:PSS)/polyvinyl pyrrolidone (PVP) films were prepared by electrospinning. In order to prepare an electrospun sandwich nanoscale film, urushiol/PAN films were deposited as both the top and bottom layers and PEDOT:PSS/PVP film as the inner layer. When the PAN to urushiol ratio was 7:5, the fiber diameter ranged between 150 nm and 200 nm. The single-layer urushiol/PAN film could not be etched after being immersed into 60%, 80%, and 100% sulfuric acid (H_2_SO_4_) for 30 min, which indicated the improved acid resistance of the PAN film. The urushiol/PAN film was used to fabricate the sandwich nanoscale films. When the sandwich film was immersed into 80% and 100% H_2_SO_4_ solutions for 30 min, the structure remained intact, and the conductive PVP film retained its original properties. Thus, the working environment tolerability of the functional PVP film was increased.

## 1. Introduction

Raw lacquer (RL) is a milky white gel-like liquid harvested from *oxicodendron vernicifluum*. It is a natural polymer-based, environmentally friendly composite material and mainly consists of urushiol (60–65%), water (20–30%), colloidal substances (5–7%), glycoprotein composition (2%), and laccase (0.2%) [1,2]. Urushiol is generally extracted from raw lacquer using ethanol and acetone as solvents. Urushiol is a derivative of catechol with long side chains of different unsaturation degrees [3,4]. Because of its special structure, urushiol has shown many excellent properties, including chemical corrosion resistance, high gloss, stable heat resistance, etc. [5,6,7]. Raw lacquer is a kind of original coating material in China, which has a history of more than 8000 years. Some ancient lacquerware is still well preserved, which shows that urushiol has excellent durability and corrosion resistance. Many researchers have done a lot of research on the performance optimization of urushiol. Deng et al. [8] prepared an excellent corrosion-resisting graphene/raw lacquer composite coating by modifying waterborne graphene with lignin tripolymer (LT; acted as an aqueous stabilizer) and subsequently adding it to RL. Zheng et al. [9] proposed a facile one-pot synthesis method for silver nanoparticles (AgNPs) encapsulated in polymeric urushiol (PUL). Silver nitrate catalyzed the polymerization of urushiol in PUL, and the antibacterial rate of the 0.1% AgNPs coating was 100% in laboratory experiments. In addition, polyamidoamine (PAMAM) is also used to improve the alkali resistance of raw lacquer. Zhang et al. [10] reported that when an RL/PAMAM film was immersed in 15% NaOH for seven days, PAMAM molecules and urushiol were cross-linked to form a dense structure, and this significantly improved the alkali resistance of the RL/PAMAM film. Jeong et al. [11] prepared urushiol powders with different amounts of 3-(trimethoxysilyl) propyl methacrylate (TPM). The as-prepared powders manifested excellent antibacterial activity, good antioxidant activity, and very high thermal stability. Cheng et al. [12] proposed an efficient and green approach to simultaneously reduce and functionalize graphene with urushiol. Urushiol with unsaturated long alkyl chains was modified on the graphene surface to disperse graphene in the organic solvent and the polymer matrix, resulting in enhanced interfacial interaction between graphene and the polymer matrix.

Polyacrylonitrile (PAN) is generally obtained from acrylonitrile extracted from petroleum through free radical polymerization. Acrylonitrile units in a macromolecular chain are connected by a head–tail method. The strength of polyacrylonitrile fibers is not high, and their abrasion resistance and fatigue resistance are poor. In addition, PAN has good weather resistance and solar resistance [13,14]. PAN nanoscale films are often used as proton exchange membranes in fuel cells [15], industrial wastewater heavy metal adsorption filtration membranes [16], and oil-water filtration membranes in marine and river environments [17]. Furthermore, PAN nanoscale films are also used in combat wear, sports, and biomedical wearable applications [13]. In order to ensure the stability of PAN nanoscale films in the aforesaid application conditions, the chemical stability of PAN is crucial.

Polyvinyl pyrrolidone (PVP) is a non-ionic polymer and is obtained through bulk polymerization, solution polymerization, and other polymerization methods using vinyl pyrrolidone (NVP) as the raw material. PVP is the most typical N-vinylamide-based polymer. As a synthetic water-soluble polymer, PVP has the characteristics of water-soluble polymeric materials, such as excellent film-forming and adhesive properties, good hygroscopicity, superb solubilization, and cohesion [18]. Lee et al. [19], developed a silver nanowire-based electrode on PVP-coated PET with low resistance and high conductivity. Zhang et al. [20] prepared precursor solutions by dissolving polymer mixtures of PAN and PVP with different weight ratios into a constant volume of dimethylformamide (DMF) through electrospinning. PVP nanoscale films are widely used in supercapacitors [21], CO_2_ adsorption [22], and lithium-ion batteries [23]. Poly(3,4-ethylenedioxythiophene) polystyrene sulfonate (PEDOT:PSS)-doped PVP is extensively used in super electrodes [24,25] and gas sensors [26] due to its better conductive effect. However, in application, PVP is not acid-resistant and is soluble in water, resulting in a short film life. Wu et al. [27] added raw lacquer to PVP and prepared nanofiber films by electrospinning, which improved the mechanical properties and acid resistance of the PVP films. However, adding raw lacquer would change some of the unique properties of PVP itself. Therefore, a coating is needed that can, not only improve the service life of the PVP film, but also maintain the PVP characteristic performance.

In the present work, urushiol was extracted from a lacquer of *oxicodendron vernicifluum* by the rotary evaporation method. Using dimethylformamide (DMF) as the solvent, different amounts of urushiol were added into a certain amount of polyacrylonitrile (PAN), and urushiol/PAN nanofilms were prepared by the electrospinning method. The addition of urushiol can improve the shortcomings of low mechanical strength and chemical stability of PAN films. The improvement of the performance of PAN films is of great significance to its application. In addition, to further explore the use of urushiol/PAN nanoscale films as protective materials, a sandwich structure of urushiol/PAN-coated PVP nanoscale film was prepared by electrospinning. This sandwich nanoscale film was immersed in sulfuric acid and pure water to investigate its corrosion resistance.

## 2. Experiment

### 2.1. Materials

Polyacrylonitrile (PAN; molecular weight = 150,000), polyvinylpyrrolidone (PVP; molecular weight = 360,000), and PEDOT:PSS were obtained from Sigma, St. Louis, MO, USA. Chinese raw lacquer (RL; Shanxi, China) was filtered with gauze. Sodium hydroxide (NaOH) and sulfuric acid (H_2_SO_4_) (Shanghai Chenghai Chemical Industry Co. Ltd., Shanghai, China) and absolute ethanol (Xilong Science Co. Ltd., Guangdong, China) were all analytically pure.

### 2.2. Preparation of Urushiol, Urushiol/PAN Mixture, and PEDOT:PSS/PVP Mixture

Preparation of urushiol: Twenty grams of anhydrous ethanol were added to 5 g of raw lacquer in a beaker and sonicated for 2 h. Impurities in the solution were filtered by a filter paper, and anhydrous ethanol in the solution was removed by the rotary evaporation extraction method at 60 °C to obtain urushiol [28].

Preparation of urushiol/PAN mixture: The preparation method of the 5:5 urushiol/PAN mixture was taken as an example. Ten grams of DMF were first added to 0.5 g of urushiol in a beaker and evenly stirred. Subsequently, 0.5 g of PAN was added to the resultant and stirred with a magnetic stirrer at 450 r/min for 8 h to obtain a mixture of urushiol/PAN. Table 1 presents different urushiol/PAN mixture ratios used in the current experiment.

Preparation of PEDOT:PSS/PVP mixture: Ten grams of DMF were added to 8 g of PEDOT:PSSS in a beaker and evenly stirred. Subsequently, 2 g of PVP was added to the resultant mixture and stirred with a magnetic stirrer at 450 r/min for 8 h to prepare a mixture of PEDOT:PSS/PVP.

### 2.3. Characterization

The IR spectra of the membranes were detected by an FT-IR spectrometer (MPIR8400S, Shimadzu, Kyoto, Japan) based on the ATR method. Sixteen scans were conducted for each film at a resolution of 4 cm^−1^. The SEM images of the as-prepared nanofilms were captured by a field-emission SEM (Nova Nano SEM 230, FEI, Hillsboro, OR, USA) at an acceleration voltage of 2 kV. The fiber size distribution (fiber diameter) was evaluated in Image-Pro Plus 6.0 software. For tensile strength (σ) measurements, each film was cut into a width (*b*) of 10 mm and a length of 100 mm and tested on a universal material testing machine (Instron 1185, Instron, Chicago, IL, USA) at a speed of 2 mm/min to record the peak load (*P*) of film fracture. The tensile strength (σ) was determined by the following formula.

(1) $$\sigma \;=\;\frac{P}{bd}$$

### 2.4. Electrospinning Process

**Figure 1:**
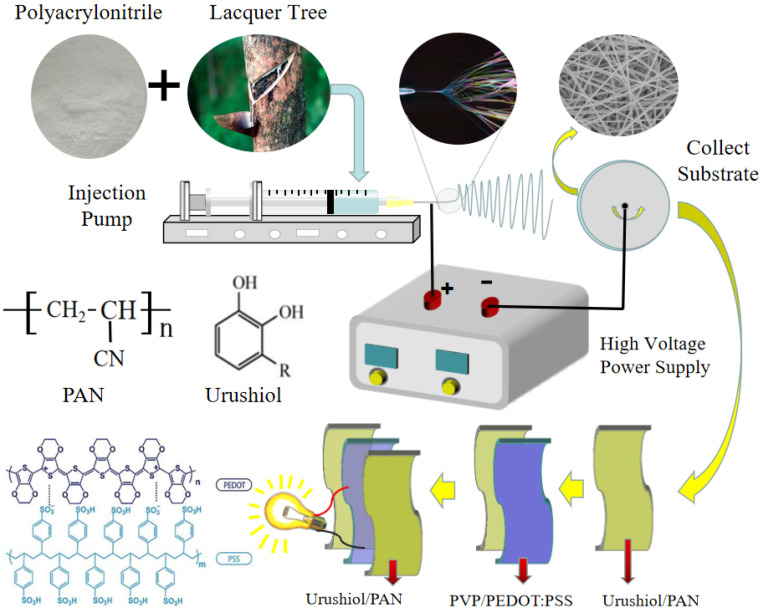
Schematic diagrams of electrospun urushiol/PAN and PEDOT:PSS/PVP nanofilms.

Preparation of urushiol/PAN and PEDOT:PSS/PVP nanofilm was completed using electrospinning equipment (JDF05, Changsha Nayi Instrument Technology Co. Ltd., Changsha, China). The mixtures were infused into a syringe. The syringe needle type was 23 G, and its inner diameter was 0.34 mm. Ten grams of dimethylformamide were used to dissolve the urushiol and PAN of different qualities, and repeated tests were conducted on different samples. Table 1 presents the average voltage values obtained from different samples. The metallic syringe needle served as the anode, while the collection board acted as the cathode, and they interacted with each other under an externally applied high voltage (Figure 1). The temperature and humidity during the experiment were strictly controlled. The humidity in the air was ≤60% at room temperature. At the feed flow rate of 0.55 mL/h, the horizontal and vertical distances between the needle tip and the collecting reel center were 14 cm and 11 cm, respectively.

## 3. Results and Discussion

### 3.1. Effects of Urushiol/PAN Ratio on Fiber Morphology

**Figure 2:**
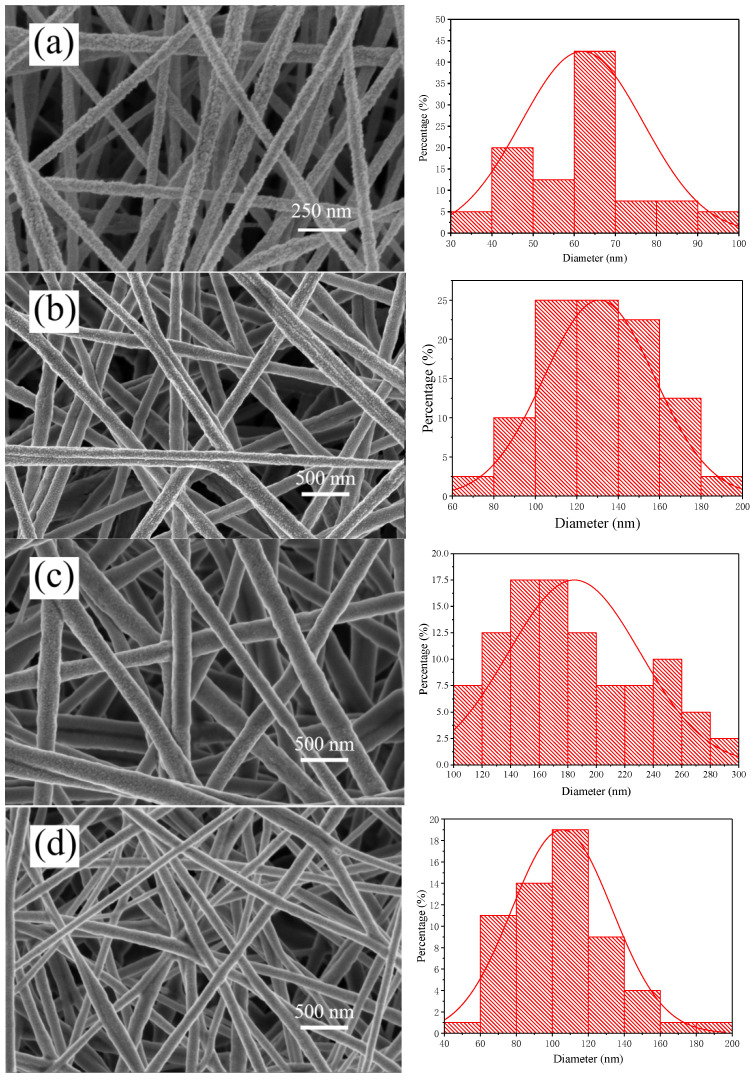

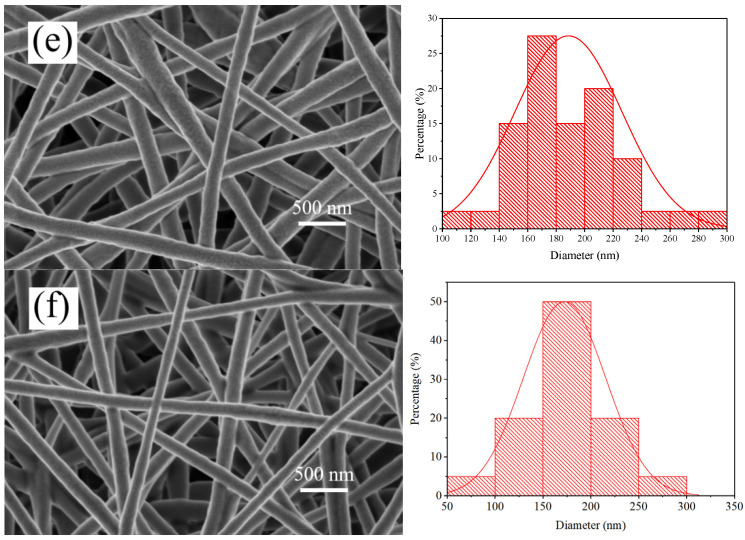
SEM images of urushiol/PAN nanofilm surfaces for different mass ratios: (**a**) Without urushiol (5% PAN), (**b**) 3:5, (**c**) 4:5, (**d**) 5:5, (**e**) 6:5, (**f**) 7:5.

Figure 2a reveals that the nanoscale PAN film prepared by electrospinning had good fiber morphology with an average fiber diameter of 40–70 nm. Figure 2b expresses that when the urushiol/PAN mixing ratio was 3:5, the fiber diameter ranged between 100 nm and 180 nm. As the viscosity and surface tension of the mixed solution increased with the addition of urushiol, the electrospinning voltage decreased accordingly, resulting in a good fiber morphology [29]. Table 1 presents the voltage values for different urushiol/PAN mixing ratios. When the urushiol/PAN mixing ratio was 6:5, the fiber diameter ranged between 140 nm and 240 nm. When the urushiol/PAN mixing ratio was 7:5 and the voltage was about 22.5 kV, the fiber diameter ranged between 100 nm and 250 nm, resulting in the optimal fiber morphology. With a further increase in the urushiol amount, voltage adjustment could not yield a good fiber morphology. When the mixture reaches the collecting substrate, the solvent evaporates rapidly, and the PAN solidifies into a fiber. Due to the long drying time of urushiol, when the urushiol content increases, it is easy to cause the undried fibers to stick together.

**Figure 3:**
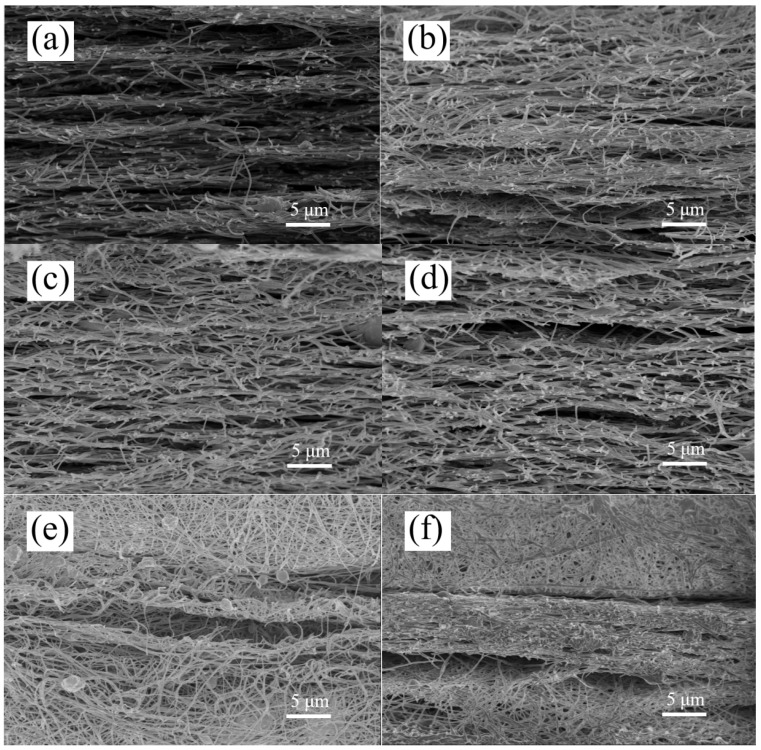
SEM images of the cross-sections of urushiol/PAN nanofilms for different mass ratios: (**a**) Without urushiol (5% PAN), (**b**) 3:5, (**c**) 4:5, (**d**) 5:5, (**e**) 6:5, (**f**) 7:5.

In order to further observe changes in the morphology of the nanoscale PAN film after urushiol addition, the cross-sectional view of the nanoscale urushiol/PAN film was observed by SEM. It is clear from Figure 3a that without urushiol, the gap between PAN film layers was large, which can be attributed to the smaller fiber diameter of the nanoscale PAN nanofilm without urushiol. As the amount of urushiol was increased, the structure of the nanoscale film became denser, manifesting a better acid-resisting performance at the same rotating speed of the collecting drum (Figure 3b–e). When the film was cut, its fibers experienced a curling phenomenon due to the brittle fracture of liquid nitrogen; however, this phenomenon was not observed when urushiol was added in a low proportion (Figure 3e,f). When the urushiol/PAN mixing ratio was 6:5 or 7:5, the fiber curling phenomenon occurred.

### 3.2. FT-IR Spectra Analysis

**Figure 4:**
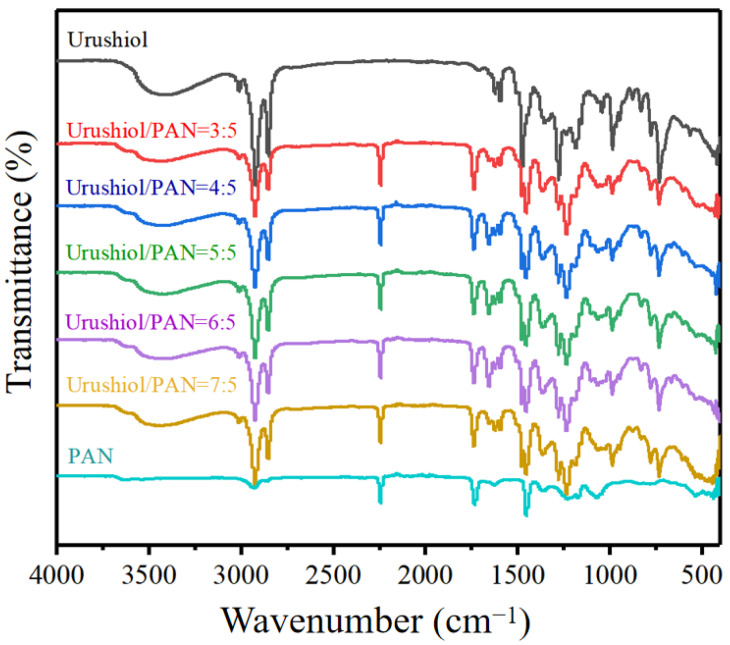
FT-IR spectra of urushiol/PAN nanofilms with different urushiol/ PAN mixing ratios.

Figure 4 displays the FT-IR spectra of the nanoscale films prepared with different urushiol/PAN mixing ratios. Urushiol is a mixture of several unsaturated branched derivatives of catechol. It contains two adjacent phenolic hydroxyl groups with the characteristics of phenol. It also has unsaturated aliphatic hydrocarbons on the benzene ring with the characteristics of unsaturated bonds. The absorption peaks of urushiol C-O-H groups at 3439 cm^−1^ and 1150 cm^−1^ were O-H stretching vibration and C-O stretching vibration [30]. The peak at 1363 cm^−1^ corresponds to the ring stretching of the aromatic carbons near the phenolic O-H, and the one at 1275 cm most probably to C-H or O-H bending. With the addition of PAN, the intensity of these peaks did not decrease, indicating that the number of O-H groups on the urushiol aromatic ring did not decrease. The vibration peaks of urushiol phenyl were observed at 1621 cm^−1^ and 1597 cm^−1^ [28], indicating that the urushiol/PAN membrane retained the structural characteristics of urushiol after adding PAN. The PAN nanofibers displayed characteristic vibrations at 2243 cm^−1^ for ν(C≡N), 2929 cm^−1^ for ν(CH_2_), and 1452 cm^−1^ for δ(CH_2_) [31]. The characteristic peak of PAN still existed after the addition of urushiol, further proving that the absorption peak and basic element of PAN were not damaged by the presence of urushiol.

### 3.3. Acid Resistance of Nanofilms

**Figure 5:**
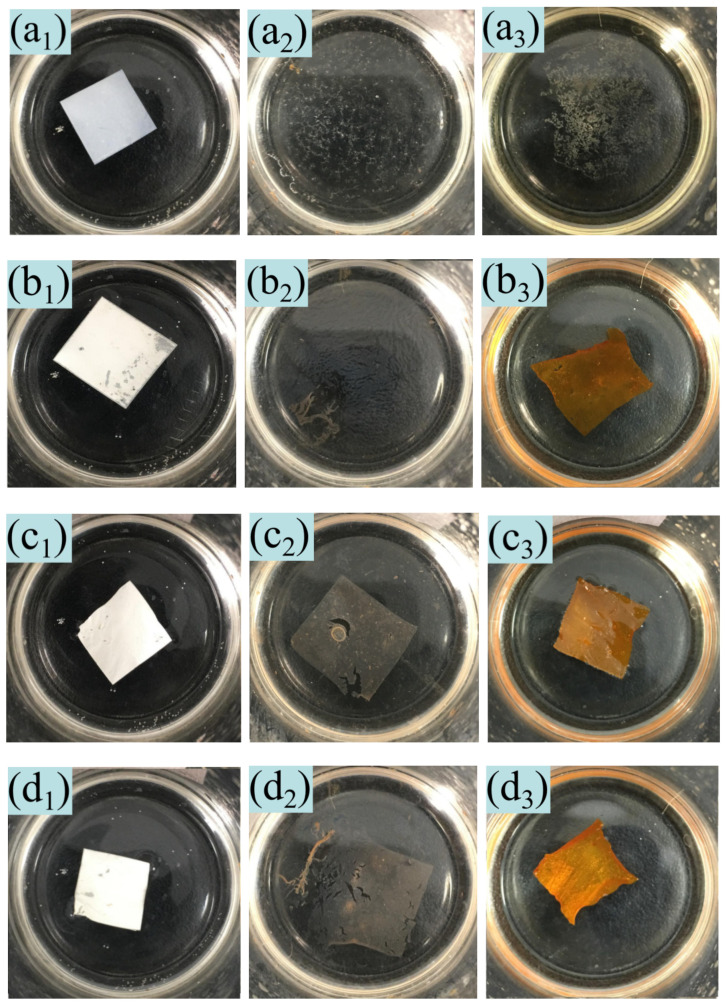

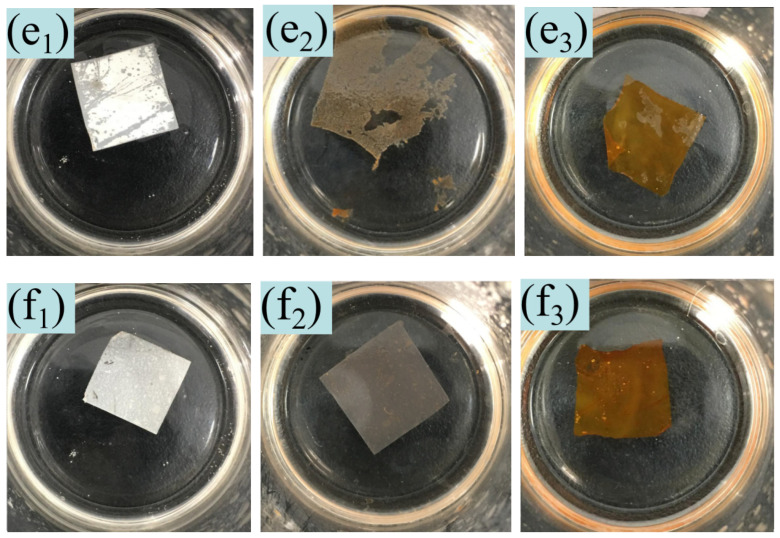
Morphologies of nanoscale urushiol/PAN films immersed in (**a_1_**) 60%, (**a_2_**) 80%, and (**a_3_**) 100% sulfuric acid solutions for 30 min: (**a**) Without urushiol (5% PAN), (**b**) 3:5, (**c**) 4:5, (**d**) 5:5, (**e**) 6:5, (**f**) 7:5.

Figure 5a displays the changes in the pure nanoscale PAN film immersed in 60% (a_1_), 80% (a_2_), and 100% (a_3_) sulfuric acid for 30 min. It is evident that the pure PAN film retained a good film-forming performance in the 60% sulfuric acid solution; however, it easily dissolved in the 80% and 100% sulfuric acid solutions. As the amount of urushiol increased, the overall film morphology in 80% and 100% sulfuric acid solutions was incomplete; however, the film morphology remained after 30 min. This is because urushiol has excellent acid resistance and the structure of urushiol will not be destroyed by electrospinning technology. The FT-IR spectra of urushiol/PAN also showed that the urushiol specific groups still existed, indicating that the urushiol/ PAN prepared by electrospinning retained the original excellent performance of urushiol. After PAN was mixed with urushiol, regardless of the mixing ratio, the film formation and morphological integrity after immersion in the 100% sulfuric acid solution for 30 min were superior to those in the 80% sulfuric acid solution. When the urushiol/PAN mixing ratio reached 7:5, the nanoscale urushiol/PAN film manifested good structural integrity in the 80% sulfuric acid solution. In order to further investigate this phenomenon, elemental analyses, and electron microscopic observations were performed on the film soaked in different sulfuric acid solutions.

### 3.4. EDS Analysis

**Figure 6:**
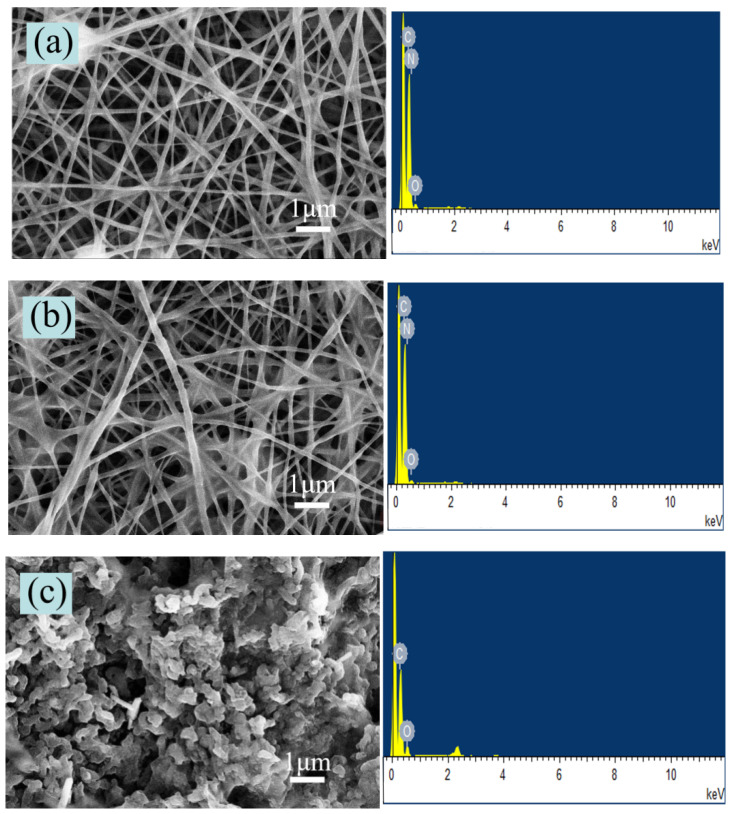
SEM images and EDS results of the 7:5 nanoscale urushiol/PAN film immersed in (**a**) 60%, (**b**) 80%, and (**c**) 100% sulfuric acid solutions.

**Figure 7:**
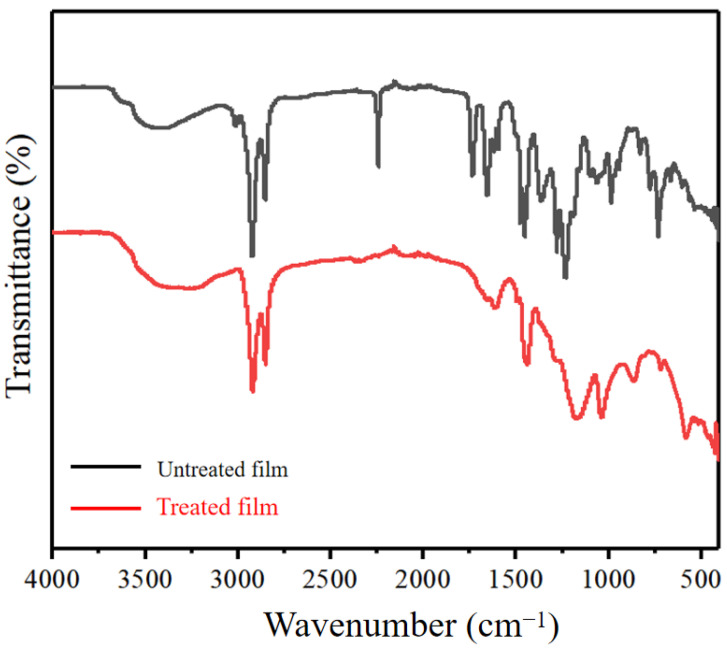
FT-IR spectra of the 7:5 nanoscale urushiol/PAN film immersed in 100% sulfuric acid solutions.

When the ratio of urushiol to PAN was less than 5:7, the film was damaged to varying degrees in 80% sulfuric acid solution. Figure 6 displays the SEM and EDS analysis results of the 7:5 nanoscale urushiol/PAN film immersed in different sulfuric acid solutions for 30 min and dried for 24 h. The acid resistance of the nanoscale PAN film after urushiol addition was greatly improved. The fiber morphology remained intact when the film was immersed in 60% and 80% sulfuric acid solutions. Table 2 presents the weight percentage changes of oxygen, nitrogen, and carbon. When the ratio of urushiol to PAN was lower than 5:7, the nanoscale urushiol/PAN film could be etched and dissolved in 80% sulfuric acid; however, urushiol/PAN film remained a complete shape in the 100% sulfuric acid solution without rupture or dissolution. The EDS analysis reveals that the nanoscale urushiol/PAN film was quickly oxidized in the strong acid solution and formed a dense oxide layer on the surface [32]. Figure 7 shows the FT-IR spectra of the urushiol/PAN film treated with 100% sulfuric acid. The characteristic absorption peaks of PAN at 2243 cm^−1^, 2929 cm^−1^, and 1640 cm^−1^ disappear. The disappearance of characteristic absorption peaks at 2243 cm^−1^, may be due to the hydrolysis of C≡N to carboxylic acid under acidic conditions. Because C=C has an electrophilic addition reaction with sulfuric acid, resulting in the disappearance of characteristic absorption peaks at 2929 cm^−1^ and 1640 cm^−1^.

### 3.5. Water Contact Angle and Tensile Strength

**Figure 8:**
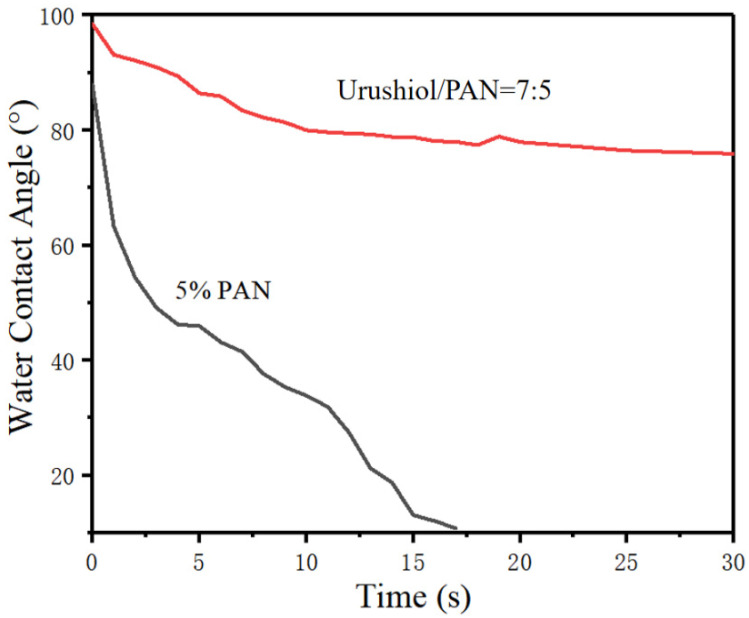
Water contact angle test results of nanoscale urushiol/PAN and PAN films.

Electrospun nanoscale films have the advantages of small fiber diameter and large specific surface area. PAN has good weather resistance and poor wear resistance. Moreover, water can easily penetrate a nanoscale PAN film. Figure 8 presents the water contact angle test results for the nanoscale PAN film. The contact angle was 0° after about 15 s, suggesting hydrophilic properties of the nanoscale PAN film. In contrast, the water contact angle of the nanoscale 7:5 urushiol/PAN film was about 80° after 15 s; therefore, the nanoscale PAN film mixed with urushiol had better water resistance. This is because urushiol is a hydrophobic material, and the addition of urushiol also showed the hydrophobicity of urushiol/PAN nanofilms. At the same time, the internal structure of the urushiol/PAN nanofilm was relatively dense after adding urushiol, which slowed down the infiltration of water molecules into the inner membrane.

The tensile strength of the nanoscale urushiol/PAN film is presented in Table 3. With the addition of urushiol, the tensile strength of the nanoscale PAN film was significantly enhanced. This is because of the high hardness of urushiol, adding urushiol can improve the mechanical properties of the film. At the same time, with the increase in urushiol proportion, the internal structure of urushiol/ PAN nanofilms became denser and denser, which was equivalent to the increase in the number of fibers per unit area.

### 3.6. A Test for Acid Resistance of Sandwich Structure

**Figure 9:**
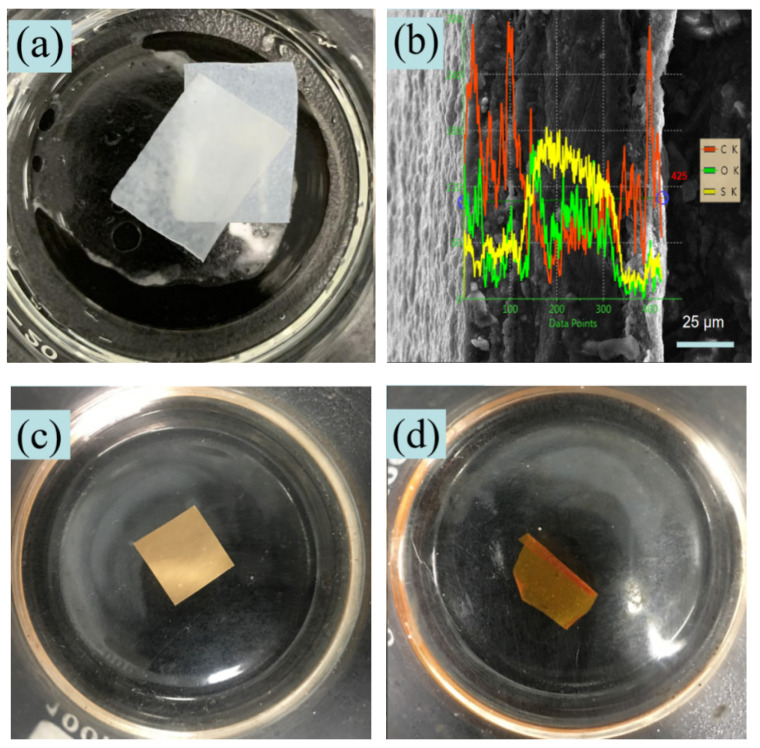
(**a**) Picture of sandwich electrospun PAN:PVP:PAN film, (**b**) EDS result of the sandwich urushiol/PAN:PEDOT:PSS/PVP:urushiol/PAN film, (**c**) picture of the urushiol/PAN:PEDOT: PSS/PVP:urushiol/PAN film immersed in the 80% sulfuric acid solution for 30 min, (**d**) picture of the urushiol/PAN:PEDOT:PSS/PVP:urushiol/PAN film immersed in the 100% sulfuric acid solution for 30 min.

Figure 9 presents the test results of the sandwich nanoscale urushiol/PAN film. Both top and bottom layers were electrospun PAN films, and the middle inner layer was electrospun PVP film (Figure 9a). PVP in the middle layer was immediately dissolved when the sandwich nanoscale film was immersed in water; thus, the nanoscale PVP film disappeared, resulting in a separation phenomenon, which limits the application of many functional nanoscale PVP films due to their hydrolysis and intolerance to acids. The SEM images of the electrospun urushiol/PAN: PEDOT:PSS/PVP:urushiol/PAN film (urushiol/PAN mixing ratio = 7:5; urushiol/PAN films were deposited at the top and bottom of a PEDOT:PSS/PVP film (inner layer)) immersed in 80% and 100% sulfuric acid solutions for 30 min are displayed in Figure 9c,d, respectively. No layer separation was observed in this case, and the overall film morphology and structure remained intact. The EDS analysis result is displayed in Figure 9b. The characteristic sulfur peak in the conductive PEDOT:PSS polymer still existed after the sandwich nanoscale film was immersed in the 100% sulfuric acid solution. The surface of the urushiol/PAN film is oxidized to form an oxide layer, preventing the acid from corroding the inner structure and hindering the etching reaction. Therefore, the electrospun 7:5 nanoscale urushiol/PAN film had good acid resistance.

## 4. Conclusions

In the current study, different amounts of urushiol were added to a fixed amount of PAN to prepare a nanoscale PAN film with good acid-resisting properties. When the urushiol and PAN mixing ratio reached 3:5, the nanoscale PAN film exhibited the same anti-etching effect as metal substances. The urushiol/PAN film could not be dissolved in the 100% sulfuric acid solution because a dense oxidized layer appeared on the film surface, preventing the acidic etching reaction with the inner layer. However, the urushiol/PAN film could be dissolved in the 80% sulfuric acid solution. As 80% sulfuric acid is less oxidizing than 100% sulfuric acid, the acidic solution could penetrate the film for the acidic etching reaction. When the urushiol and PAN mixing ratio was 7:5, a superior acid resistance effect was noticed, and the film remained intact in either 80% or 100% sulfuric acid solutions. Therefore, the urushiol/PAN mixing ratio of 7:5 was used to protect the nanoscale PVP film. In order to prepare an electrospun sandwich nanoscale film, urushiol/PAN films were deposited as top and bottom layers on a PEDOT:PSS/PVP film (inner layer). After the immersion of the nanoscale PEDOT:PSS/PVP film in 80% and 100% sulfuric acid solutions, the sulfur peak of PEDOT:PSS was still noticed, demonstrating good acid resistance of the urushiol/PAN film. When urushiol was added in a low proportion, PAN yielded an acidic reaction between 80% and 100% sulfuric acid solutions. Therefore, urushiol/PAN films can be used as unique materials with strong acid resistance and weak acid dissolution properties in the future.

## Figures and Tables

**Table 1:** Voltage values of urushiol/polyacrylonitrile (PAN) and Poly(3,4-ethylenedioxythiophene):poly(styrenesulfonate)(PEDOT:PSS)/polyvinyl pyrrolidone (PVP) films prepared with different urushiol/PAN mixing ratios.

**Urushiol/PAN (g)**	**Voltage 1 (kV)**	**Voltage 2 (kV)**	**Voltage 3 (kV)**	**The Average (kV)**
5% PAN	29.54	27.32	27.00	27.95
3:5	23.61	27.08	26.08	25.59
4:5	23.65	23.53	25.02	24.06
5:5	23.31	23.85	23.44	23.53
6:5	23.05	23.25	22.96	23.09
7:5	22.31	22.36	22.79	22.49
**PEDOT:PSS/PVP (g)**	**Voltage 1 (kV)**	**Voltage 2 (kV)**	**Voltage 3 (kV)**	**The Average(kV)**
4:1	27.51	28.07	27.68	27.75

**Table 2:** EDS results of the 7:5 nanoscale urushiol/PAN film immersed in different concentrations of sulfuric acid.

Percentage by Weight	C K	N K	O K
60%	66.89	23.11	10.00
80%	71.10	20.20	8.70
100%	76.74	0	23.26

**Table 3:** Tensile strength results of urushiol/PAN nanofilms.

Urushiol/PAN (g)	Wide (mm)	Thickness (mm)	Peak Load (N)	Tensile Strength (MPa)
5% PAN	9	2.817 × 10^−1^	1.751	0.691
3:5	9	9.254 × 10^−2^	0.850	0.856
4:5	9	1.371 × 10^−1^	2.551	2.067
5:5	9	8.058 × 10^−2^	1.950	2.689
6:5	9	9.106 × 10^−2^	2.651	3.235
7:5	9	4.506 × 10^−2^	1.751	4.318
